# Dr. Adams, on Vaccination

**Published:** 1805-01-01

**Authors:** Joseph Adams

**Affiliations:** London


### Medical and Phyfical Journal.

VOL. XIII.] JANUARY 1, 1805. [NO. LXXI.

*Printed for R. PHILLIPS, by W. Thorne, Red Lion Court, Fleet Street, London.*

### To Dr. BRADLEY.

DEAR SIR,

DURING my late retirement at New Galloway, your Numbers arrived so slowly, and so irregularly, that I was not aware till my return to London of the notice with which two of your Correspondents had honoured me.

To Dr. Walker's remarks I have but little to say, as we seem not perfectly to understand each other. "If, says that gentleman, vaccine lymph can, under any circumstances, produce a general eruption: in such departure from its original character we have, perhaps, one first step of vacciola to its degenerated state of variola." By this are we to understand, that any doubt can remain that the vaccine inoculation is sometimes attended with a general or secondary eruption? Does any one doubt the cases related by so many experienced practitioners in England, and in other parts of Europe? Lastly, do they doubt, quod *ipse dixit*? Does not Dr. Jenner speak of eruptions succeeding the vaccine inoculation, under certain circumstances?

Dr. Walker afterwards remarks, that he has never noticed those varieties pointed out by me. This is very probable; but it should be remembered, that no one but Norris ever remarked the suspension of inoculation for small-pox by the Harmattan wind.

Dr. Walker suspects my conclusion, "that the eruptions in question arose from cow-pock matter, may, after all, have no better foundation than mere hypothesis." On this occasion I am satisfied to stand on the same ground as Dr. Woodville, who, as well as myself, inoculated with secondary vesicles, and produced the genuine cow-pox.

I am extremely glad to be set to rights in the last paragraph of this communication, and to find not only that the Jennerian Society have established a centre of communication for receiving information on the subject of cow-pox, but also that their letters are post free. I cannot however help being surprised that this indulgence has not been extended to Dr. Jenner. The number of communications received by that gentleman must be still very considerable, because many people, like myself, will be ignorant of the committee and its privileges.

Mr. Ring's, as well as Dr. Walker's, exordium consists of a compliment. It is pleasing to see this urbanity among men who differ in opinion, and, indeed, who are scarcely known to each other but by this difference. It is very true, Mr. Ring, when we met previous to my journey into Scotland, mentioned not only his doubts relative to the vaccine eruptions which occurred in Madeira, but also his suspicions that they were variolous. I shall at present only observe, that of between twenty and thirty children, who had intercourse with the patients, none were casually infected, though all of them, when inoculated in classes, had the genuine cow-pox without secondary eruptions.

Mr. Ring remarks, that he has seen many such eruptions, but never saw any that resembled vaccine pustules. If by this is meant a resemblance to the inoculated part, we perfectly agree. But, adds Mr. Ring, *matter* taken from them has produced the chicken-pox. This is definitive as to this part of the controversy; the vesicles I speak of contained no *matter*, only a transparent lymph.

Mr. Ring's concluding remarks are most of them very just. Anonymous publications on a subject so important to human life and the tranquillity of thousands, should be considered as firebrands thrown at random, and should be instantly, and without inquiry, stifled. But communications from men of character, however imperfect, should be always attended to. We not only inform others by receiving and answering them, but we improve our own stock of knowledge, and by degrees shall learn an accuracy of discrimination which is still unattained. Add to this, the alarm is much lessened, when a subject is candidly heard, and the relater treated like a reasonable being, than when he is rudely contradicted or undervalued. Let us attend to the manner in which Mr. Cardan, of Worcester, treated a family who were prepared to calumniate him, and alarm a whole neighbourhood. A child, whom he had inoculated for cow-pox, had afterwards the small-pox. Whether there had been any irregularity in the first insertion would have been a very unimportant inquiry to the mother. This judicious practitioner therefore, without hesitation, admitted ted the fact; observing, that the same thing sometimes, tho' rarely, happened in the small-pox after inoculation, or even when received in the casual way; that such an event was not to be generally expected, because the small-pox was rife through the neighbourhood, and few escaped who had not been inoculated with cow-pox; yet of the latter, this child alone had taken the disease. If, however, the neighbourhood were still alarmed, Mr. Cardan expressed his intention of being among them on the following day, when he should inoculate with small-pox all such as wished for it, and that all of them might have an opportunity of exposing themselves and their children to the effluvia of that disease. This fair proposal, joined to the conciliating manner in which it was made, quieted every suspicion; few gave themselves the trouble to inoculate, all exposed themselves without reserve, and all remained free from any variolous symptoms.

I shall now offer a few remarks on a very valuable paper by Dr. Macdonald, in answer to Mr. Goldson's pamphlet. It is most sincerely to be wished, that in all our controversies we could learn to give our antagonist credit for all the good intentions he professes. A very slight knowledge of the world will teach us, that this is the readiest way to convince him. If our wish is to convince the world, we should recollect that the *argumentum ad hominem* never fails to tell as much on one side as the other. If the favourers of vaccination should be ready to admit some of those motives, suspected by Dr. Macdonald, its enemies will think still worse of a cause which requires such insinuations to support it.

It is not my intention to go through the various statements and arguments with which this communication abounds. I am as well convinced as the author, that most if not all of Mr. Goldson's cases were attended with some irregularity, or some defect of evidence, which renders them unfit to form any decisive conclusion; but we should recollect that this very defect or acknowledged irregularity reflects the highest honour on Mr. Goldson. The following passage from Dr. Macdonald is transcribed, in order to shew his description of small-pox, and to draw what appears to me a fair conclusion from his inference.

" As these (Mr. Goldson's) cases never maturated, they never could have passed through their regular stages, so well marked in the small-pox, even in its mildest form. About the third or fourth day, eruptions make their appearance; pearance; first, in the form of small red spots, hardly elevated. These gradually increase in size, and after two or three days more, form a small vesicle, which is surrounded by a circular inflamed margin, and contains a clear, limpid fluid. For the three or four succeeding days the pustules become larger, more elevated, and acquire their proper figure and size. At the end of this period, the clear limpid fluid contained in the vesicle is changed into a purulent matter, which is formed into a crust on its surface; and, lastly, after some time, these crusts fall off, and leave reddish brown spots on the skin below. Had any of Mr. Goldson's cases gone through such a regular course as this, I believe he would have done so much justice to his own cause, as not to have omitted that circumstance."—See Dr. Macdonald's paper, p. 321.

Can we have a stronger proof of Mr. Goldson's veracity, than that, when he was anxious to discredit vaccination, he should strain none of his evidence so far as to describe "that regular course," which must have silenced every objection? On the contrary, we find him describing no other course of symptoms than such as Dr. Macdonald sees no difficulty in proving, could not be variolous. I am aware it will be said, that though Mr. Goldson's facts are not decisive to the purpose for which he states them; yet, that he takes much pains to make them appear so. To this I answer, that we are by no means aware how much we are all warped by our own preconceptions, and that the utmost we can expect of each other is to confine ourselves to truth. Mr. Ring tells me, in "the paper above alluded to, that I shall not call Dr. Willan a zealot." To this I answer, that I call myself one; and the ardour with which Dr. Willan has pursued his inquiries, seems to entitle him to that epithet. It is this consciousness of zeal in ourselves that should induce us to make large allowances for each other's failings, and, like Mr. Goldson, to be particularly cautious in the statement of our facts, though they should be insufficient to insure those inferences we may wish to draw.

We shall however presently find, that Dr. Macdonald's description of small-pox is not always to be depended on. "But, (says he, p. 323) independent of this, the number of pustules and their duration by no means prove that it was the small-pox; I have frequently witnessed cases of varicella, where the pustules were as numerous, lasted as long, and left marks behind like the small-pox. Lest my authority should be deemed insufficient, I shall quote that of an abler abler judge. Mr. Ring, in his Treatise on Cow-pox, p. 829, mentions, that many people have seen the chicken-pox in some measure confluent, and that he himself has known a considerable number leave a cicatrix behind. In p. 895, he mentions the case of a child, in which the eruption was not complete until the tenth day; and the last vesicle did not disappear till the fourteenth." After this follow a number of instances, in which impostor drug-shop sweepers and respectable practitioners have deceived others, or been deceived themselves, not only in our own days, but in the days of older authors. "Both Cullen and Heberden, it is added, maintain, that *medical men* were often deceived by the chicken-pox, from its assuming the appearance of the small-pox." This subject occupies three or four pages, at the end of which, Dr. Macdonald, expressing a due deference to superior judgment, confesses himself sceptically inclined when cases are related of persons having small-pox twice, adding, that this opinion is not new any more than the uncertain discrimination between the chicken and small-pox.

That such doubts, and such uncertainties, should have existed formerly, is not very wonderful; but that, in the present day, we should be inquiring after a never-failing character of the small-pox, is not less surprising to me than if, at noon, we should be inquiring for the sun, when the sky is unclouded.

We have been anciently reminded, that we are of more value than the herb of the field, which to day is, and to morrow is cast into the oven. Were I to doubt such a position, who would not be justly offended? But I will be bold to say, that Botany is pursued with an ardor, and cultivated with an accuracy, to which Physiology, or rather Pathology, is a stranger. How well do I recollect the eagerness with which a botanist has searched for the nectarium attached to the bottom of the petal, in order to fix the genus Ranunculus. With what triumph have I seen a zealous disciple of Linnæus demonstrate to an Hudsonian this unequivocal character in what the latter called Ficaria verna. Each party had his never-failing marks to exhibit; to these each could appeal: and if they were neither disposed to be convinced, neither could dispute the evidence of his senses. Will it be believed, that in discriminating the small-pox we have marks as regular, as certain, as constant, and as unequivocal, to distinguish it from every other eruption, as the nectarium which characterizes the genus ranunculus? Will it be believed that this mark has

 never never been sought for, nor hinted at, in all the controversy that the important discovery of cow-pox has introduced?

In one of the volumes of the Philosophical Transactions, Mr. Hunter gives a case of small-pox in a child at its birth. To ascertain whether the disease was really small-pox, he dissected several of the pustules, and in each of them found the slough which lines the bottom of the pustules, and in some instances forms the future pitting. This, he assures us, is the true character of the small-pox, being constant in every pustule. The chicken-pox, on the contrary, is free from this appearance, the bottom never sloughing excepting in a few pustules where inflammation is particularly high. It is true, the Philosophical Transactions are not in every library, but when engaged in controversy, it becomes us to examine every source of information. Mr. Hunter has also mentioned this slough in the Introduction to his Treatise on the Blood, observing, that how slight soever the inflammation may be, it always induces slough. I should not have presumed to mention my own name after Mr. Hunter had been passed by, had it not been that some of the gentlemen alluded to, have done me the honour to quote me. In "Morbid Poisons" I have taken great care to describe this slough, when speaking of small-pox; and in the paper which Mr. Ring quotes from your Journal of April, 1803, I remarked, that my first business, after viewing my eruptive cases, was to search for the slough.*

Before I described this slough in 'Morbid Poisons,' not thinking it sufficient to avail myself of Mr. Hunter's name, however respectable, it was my business to search for it, and afterwards to demonstrate it to the two apothecaries then resident at the Small-pox Hospital. I pointed it out to Mr. Pitcairn, at that time my pupil; and have lately induced Mr. King, of Clifton, to search for it, which he did to his own satisfaction.

In a letter to Dr. Clutterbuck, inserted in one of your Journals, the attention of the reader was particularly called to the slough; yet, though these controversies have continued for more than two years since that time, no writer has taken the trouble to dissect a pustule, or to avail himself of this only distinguishing characteristic of the disease. I should

---

* I wish it to be remarked, that this, and every other quotation excepting from your Journal, No. 68, is from memory, having at present no means of referring to my library.

should be ashamed of again reverting to the subject, were it not that the acknowledged want of any other certain diagnosis of small-pox renders it absolutely necessary. Let me therefore conclude with entreating, that whenever any future doubts are entertained of this disease, those who are disposed to write on the subject will dissect the pustules about the time they become purulent, before they scab; and at the bottom of each will be found a loose slough of a circular form, about the thickness of writing paper, which it resembles when soaked. If this is found, the disease is small-pox; if it is not found, the disease is not small-pox.

